# Post-extraction volumetric analysis of alveolar ridge contour using subepithelial connective tissue graft in esthetic zone: a randomized controlled clinical trial

**DOI:** 10.1007/s00784-023-05255-0

**Published:** 2023-09-19

**Authors:** Nourhan Gamal, Nesma Shemais, Marwa Al-Nawawy, Noha A. Ghallab

**Affiliations:** Oral Medicine and Periodontology, Faculty of Dentistry, Cairo University, Giza, Egypt

**Keywords:** Connective tissue graft, SCTG, Alveolar ridge contour, Pouch technique, Post-extraction dimensional changes, Linear volumetric change

## Abstract

**Objectives:**

The aim of this randomized clinical trial was to assess the alveolar ridge contour after soft tissue augmentation using subepithelial connective tissue graft (SCTG) buccal to fresh extraction sockets in patients with thin buccal bone, versus minimally-traumatic extraction followed by spontaneous healing solely.

**Materials and methods:**

Forty non-restorable maxillary teeth in the esthetic zone were randomly assigned into two groups: minimally-traumatic extraction with SCTG (test) and minimally-traumatic extraction followed by spontaneous healing (control). The outcomes assessed included linear volumetric change of buccal soft tissue contour, vertical tissue loss, gingival thickness (GT), and interdental papilla (IDP) height after 6 months.

**Results:**

The SCTG group showed a significant improvement (*P* < 0.001) in all outcomes after 6 months. The SCTG group showed a statistically significant (*P* < 0.001) gain in the buccal soft tissue volumetric change compared to the control group. The SCTG group showed a statistically significant increase in GT (*P* < 0.001) and IDP height (*P* < 0.05) after 6 months compared to the control group.

**Conclusions:**

The use of SCTG buccal to extraction sockets in the anterior maxilla might be considered as a predictable approach for preserving the alveolar ridge contour.

**Clinical relevance:**

SCTG buccal to extraction sockets might counteract post-extraction hard and soft tissue alterations in the esthetic zone.

## Introduction

Following tooth extraction, several sequential events arise causing significant qualitative and quantitative alterations at the edentulous site. Socket healing process results in ridge dimensional changes of the underlying bone, as well as the overlying soft tissue architecture [1]. Loss of soft tissue contour following tooth extraction could persist after healing, regardless of the utilization of alveolar ridge preservation techniques. As a result, clinicians seek different surgical techniques to restore the post-extraction hard and soft tissue volume loss [2]. Since esthetics is the main concern for most patients, soft tissue grafting is becoming a routine in the daily clinical practice [2, 3], to compensate for the diminished supra-crestal tissue dimension that usually occurs following tooth loss and implant placement [4]. Such procedures have a role in increasing tissue thickness, re-establishing an adequate width of keratinized tissue, correcting mucogingival deformities, and improving esthetics around teeth and dental implants [5–7]. Moreover, the presence of a sufficient quantity and quality of soft tissues play a chief role in the long-term maintenance of peri-implant health [8]. However, recent retrospective study reported no difference in volumetric, linear changes, and peri-implant conditions between implant sites with or without soft tissue grafting over a period of 12 years [9].

The concept of performing soft tissue augmentation on the buccal side of the extraction socket following tooth extraction was adopted by many experts and clinicians, in order to stabilize the soft tissues and compensate for the buccal concavity that arises after tooth loss [10]. Meanwhile, there is an extensive body of evidence proving that subepithelial connective tissue grafts (SCTGs) are considered the gold standard in ridge contour augmentation procedures [11–14]. Previous review articles and systematic reviews concluded that various therapeutic approaches are built on SCTG-based procedures and that SCTG is superior in soft tissue correction and augmentation surgeries [13, 15, 16]. This is in agreement with the conclusions of the latest consensus report of group 2 of the SEPA/DGI/OF workshop [17]. The authors stated that superior esthetic outcomes were observed in the presence of a thick mucosa; the connective tissue graft remains the standard protocol of care in terms of increasing mucosal thickness.

Based on the current available literature, clinical research related to dimensional alterations post tooth extraction mainly focused on the hard tissue modeling, while the impact of soft tissue healing in post-extraction sites received a little consideration. The investigation of post-extraction soft tissue volumetric changes in future clinical trials was strongly recommended in the latest consensus report on the management of the extraction sockets and timing of implant placement [18]. Given the existing gap of knowledge, this randomized clinical trial aimed to volumetrically assess the alveolar ridge contour after soft tissue augmentation using SCTG buccal to fresh extraction sockets in patients with thin buccal bone, versus minimally-traumatic extraction followed by spontaneous healing with no soft tissue augmentation procedure.

## Materials and methods

### Study population

**Fig. 1:**
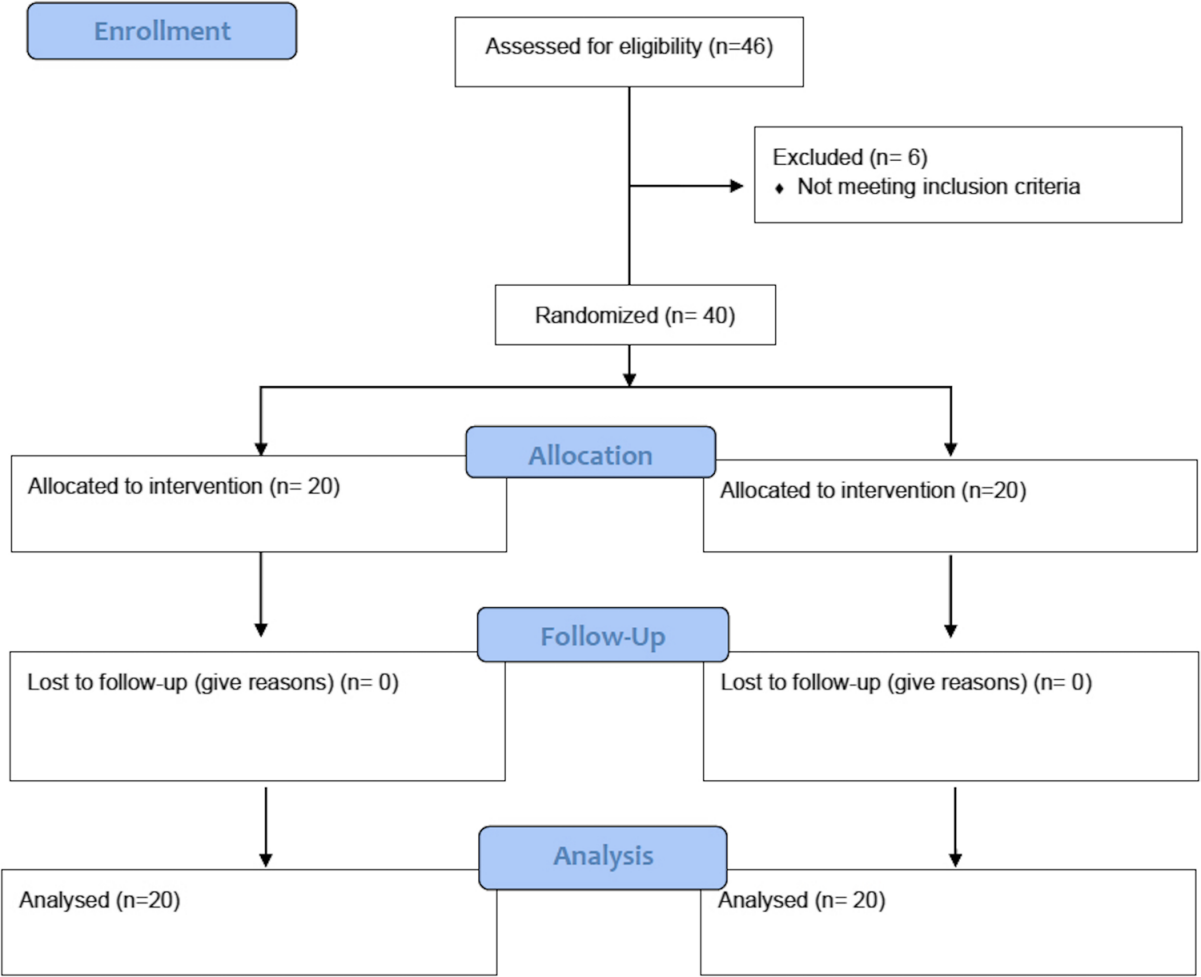
CONSORT flowchart

The present randomized clinical trial was registered in the ClinicalTrials.gov (ID NCT04482127), approved by the Research Ethics Committee, Faculty of Dentistry, Cairo University (13–12-19), conducted in accordance with the Helsinki Declaration of 1975, as revised in 2013 and reported according to the CONSORT guidelines [19] presented in Fig. 1. This study included 20 non-restorable maxillary teeth indicated for extraction in the esthetic zone in 32 patients selected from the outpatient clinic, Department of Periodontology, Faculty of Dentistry, Cairo University, between January 2020 and March 2022. The sites included 5 central incisors, 4 lateral incisors, 3 canines, and 8 premolars in SCTG group and 3 central incisors, 4 lateral incisors, 3 canines, and 10 premolars in minimally-traumatic extraction followed by spontaneous healing group. All participants met the following inclusion criteria: having non-restorable maxillary teeth indicated for extraction from 2nd premolar to 2nd premolar region, intact gingival tissue with at least 2-mm keratinized tissue, buccal bone thickness 1 mm or less assessed by CBCT^1^ preoperatively, and periodontally and systemically healthy patients. Exclusion criteria included pregnant and lactating females, current and former smokers, and presence of active infection with soft tissue communication. All patients provided written informed consent to participate in this trial. Initial patient examination was performed including full-mouth supragingival scaling and 0.12% chlorhexidine HCL^2^ mouthwash twice daily was prescribed for 2 weeks with patient motivation and oral hygiene instructions.

### Randomization and blinding

Sequence generation was executed using simple randomization by a computer-generated list from numbers 1:40 by an investigator (NM) who was not involved in recruitment nor treatment. Allocation concealment was implemented by the same investigator using sequentially numbered, opaque, sealed envelopes handled to the surgeon (GN) who did not open them until the beginning of the interventions. After pretreatment phase, eligible participants were randomly assigned into two parallel groups with a 1:1 allocation ratio to receive either SCTG following atraumatic extraction (test group) or minimally-traumatic extraction followed by spontaneous healing only (control group) based on the generated sequence. Due to the difference in the intervention’s techniques, both the operating surgeon and the participants could not be blinded to the procedure. The outcome assessor (SN) and statistician (GNA) were blinded.

### Volumetric analysis

The primary outcome in this clinical trial was the postoperative linear volumetric change of buccal soft tissue contour. Conventional polyvinyl siloxane impressions^3^ were taken at baseline, 3, and 6-month postoperative for each patient. The stone casts produced from the impressions were optically scanned using lab scanner^4^ to create digital surface models in Standard Triangle Language (STL) format, all STL files were imported to a digital software.^5^ The best-fit algorithm was used to superimpose digital surface models, when comparing each area of interest (AOI) throughout out the follow-up period. Unchanged neighboring teeth surfaces were used as a reference for proper superimposition. In each patient, the AOI was kept constant for all pairwise comparisons. The volumetric analysis software calculated a mean linear volumetric change (mm) within the AOI for each patient at baseline, 3 months, and 6 months postoperative.

### Clinical parameters

Clinical parameters were recorded at baseline and 3 and 6 months postoperatively by a single examiner (SN) who was blinded, trained, and calibrated with a good intra-examiner agreement (0.82 ĸ value). Clinical parameters recorded were gingival thickness (GT) and interdental papilla (IDP) height as secondary outcomes. GT measurements were recorded using anesthetic needle with a stopper technique [20], at levels of 1.5 mm, 3 mm, and 4.5 mm from the free gingival margin. The IDP height was recorded using UNC15 periodontal probe from the base to the tip of the IDP; the reference was the anatomical marks of the preoperative interdental papilla and the adjacent teeth. Accordingly, postoperative measurements were done using the UNC15 periodontal probe. A 6-month postoperative evaluation period was chosen based on the recommendation that final restorative measures should not be initiated until 6 months after ridge augmentation procedures. Furthermore, qualitative and probably quantitative alterations can arise during the healing period of the augmented soft tissue [21, 22].

### Treatment protocol

#### Minimally-traumatic extraction

In both groups, flapless minimally-traumatic extraction was performed using peristomes^6^ inserted along the root surface; apical pressure and rocking motion were applied circumferentially to cut the periodontal ligaments. After initial luxation using Harpoon luxators,7 bayonet forceps were used to deliver the tooth out of the socket. Socket debridement was done to make sure the socket was thoroughly clean. For the control group, minimally-traumatic extraction followed by spontaneous healing was performed without soft tissue augmentation procedure.

#### Recipient site preparation (pouch technique + SCTG)

**Fig. 2:**
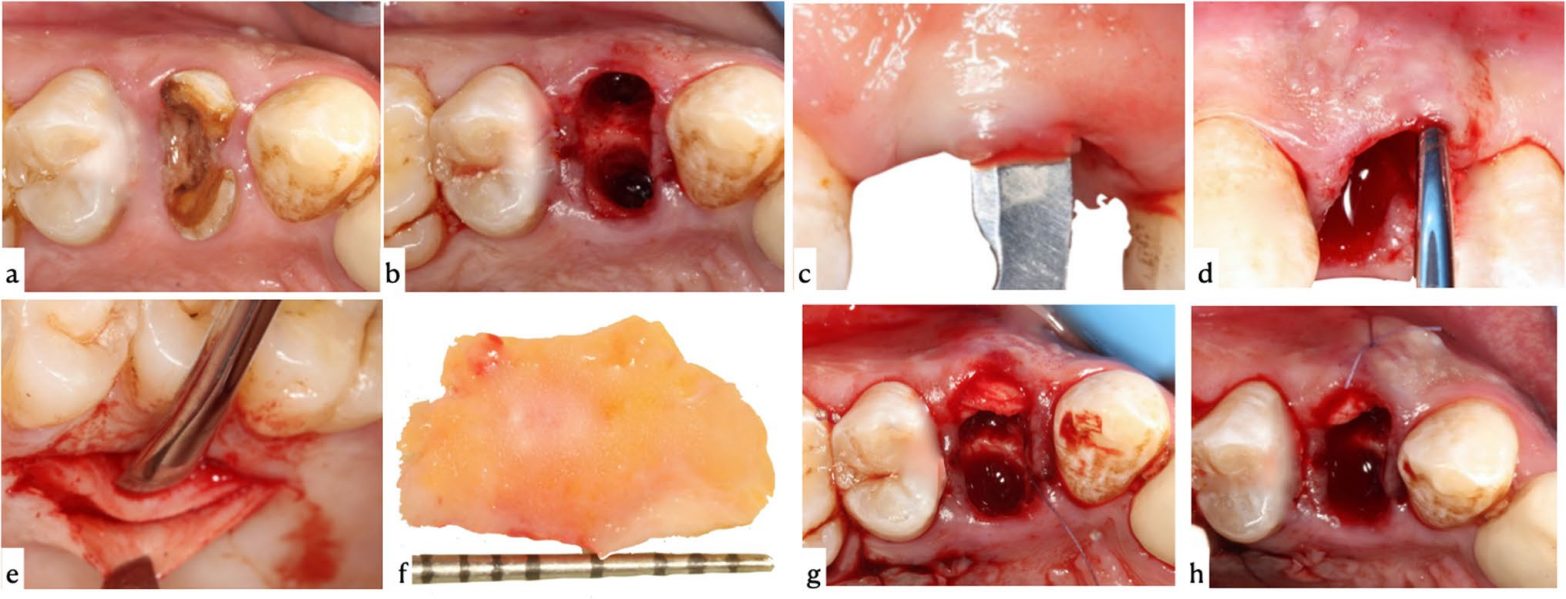
Overview of minimally-traumatic extraction + SCTG. **a** Preoperative clinical photograph occlusal view. **b **Minimally-traumatic extraction. **c** Initial incision of split thickness buccal pouch. **d** Extension of pouch preparation using tunneling knife. **e** SCTG harvesting from the underlying bone by blunt dissection. **f** Harvested SCTG. **g** SCTG fitted in the buccal pouch. **h** SCTG secured in desired position by periosteal suture

Patients assigned to the intervention group received pouch technique + SCTG represented in Fig. 2. Following minimally-traumatic extraction, preparation of a split thickness buccal pouch between the buccal bone plate and the overlying gingiva of the extraction socket was performed using 15c mini blade^7^ and tunneling knifes.^8^ Initial partial dissection incision was done with an angle directed towards the buccal bone to avoid perforation of the overlying flap. The pouch preparation was then extended apical to the mucogingival junction and to the neighboring teeth mesiodistally using the tunneling knife to ensure sufficient flap mobility. The SCTG was harvested from the palate with a standardized thickness of 1.5 mm using single incision technique [23], which was done approximately 2–3 mm apical to the gingival margin of the maxillary teeth and parallel to the palatal long axis. Partial thickness flap was then raised and SCTG was separated by four down-to-bone incisions and harvested from the underlying bone by blunt dissection. The SCTG was contoured to fit the prepared pouch and then inserted and secured into the recipient site by a periosteal suture from the inside of the prepared pouch, using a resorbable suture material.^8^

### Postsurgical phase

Postoperative analgesics^9^ three times daily and systemic antibiotics^10^ twice daily were prescribed for 5 days. Patients were instructed to rinse with 0.12% chlorhexidine HCL2 three times a day for 2 weeks and to avoid any hard brushing and trauma to the surgical site. Cold ice packs were recommended to be placed extraorally adjacent to the surgical area during the first 12 h. For the test group, sutures were removed 14 days post-surgically.

### Statistical and power analysis

Sample size calculation was based on finding a mean difference of at least 0.5 mm between the two studied groups with SD of 0.5 mm based on previous data [24]. Alpha was set at 0.05 and the power was set at 0.8. This resulted in the inclusion of 17 sites per group. To compensate for possible dropouts, 20 sites were included in each group, with a total of 40 sites. The sample size was calculated by PS program.^11^ Data was explored for normality using Kolmogorov–Smirnov test and Shapiro–Wilk test. Continuous data showed normal distribution and were described using mean, standard deviation, mean difference, and 95% confidence interval. Repeated measures ANOVA test was used for comparisons between and within groups followed by Tukey post hoc test. Tukey post hoc test was used for pairwise comparisons when ANOVA test was significant. A *P* value less than or equal to 0.05 was considered statistically significant and all tests were two tailed. Data was analyzed using MedCalc software, version 19 for windows.^12^

## Results

### Initial buccal bone thickness

The SCTG group presented an initial buccal bone thickness with a mean of 0.75 ± 0.13, while the minimally-traumatic extraction followed by spontaneous healing group showed a mean of 0.77 ± 0.15. Intergroup analysis revealed a mean difference [95% CI] of − 0.021 [− 0.12, 0.07], with no statistically significance (*P* value = 0.65) between the two groups regarding the baseline bone thickness.

### Volumetric analysis

#### Buccal soft tissue contour

**Fig. 3:**
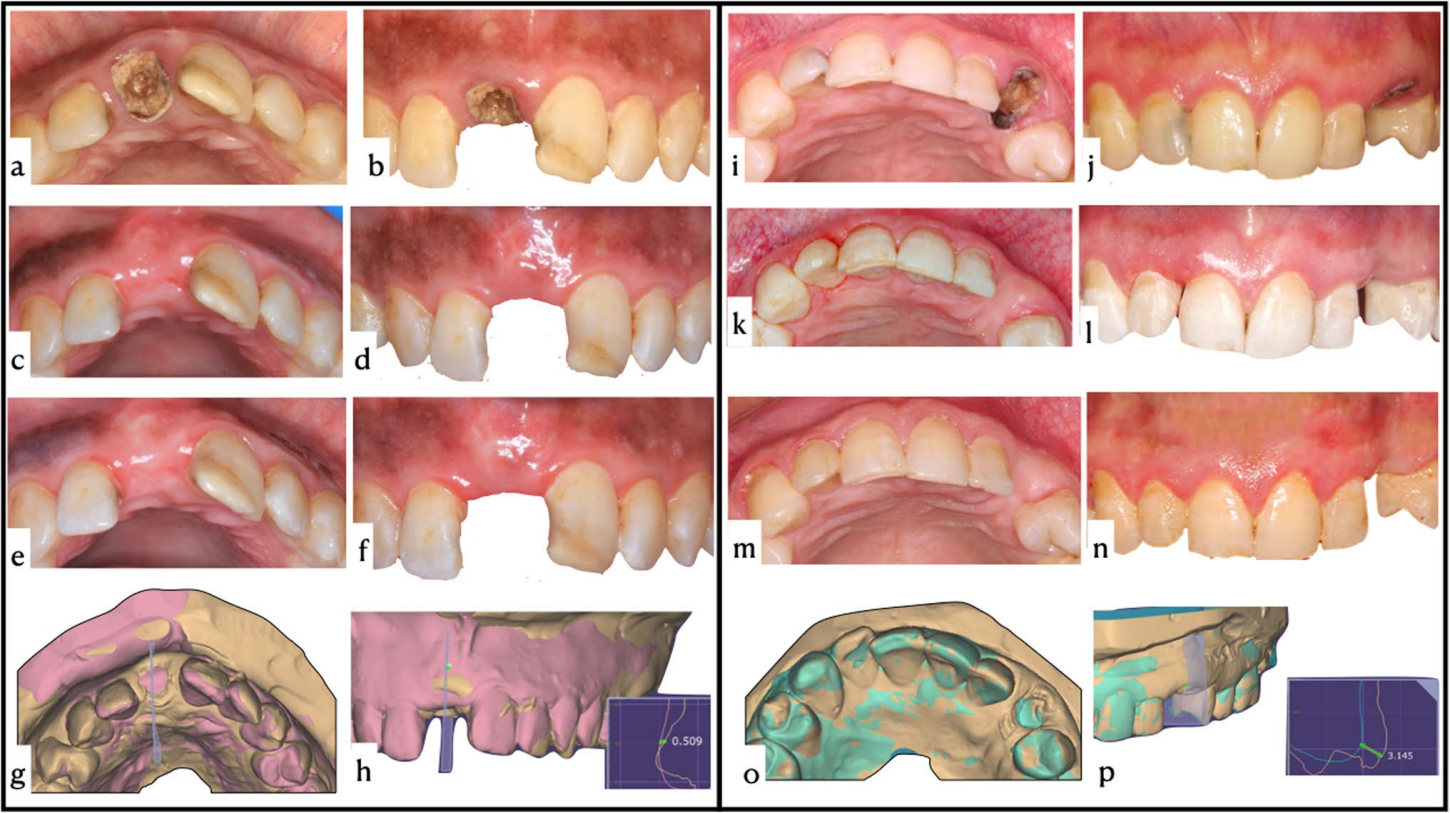
Case description including preoperative, 3 months, and 6 months postoperative clinical photographs and 0–6 months linear volumetric change of test group (from **a** to **h**) and control group (from **i** to **p**). **a** Preoperative clinical photograph, occlusal view, **b** preoperative clinical photograph, frontal view, **c** 3 months postoperative clinical photograph showing soft tissue contour, **d** 3 months postoperative clinical photograph showing IDP heights and vertical tissue level, **e** 6 months postoperative clinical photograph showing soft tissue contour, **f** 6 months postoperative clinical photograph showing IDP heights and vertical tissue level, **g** 0–6 months superimposed models by best fit algorithm, **h** 0–6 months superimposed models showing gain of 0.5 mm in buccal soft tissue contour, **i** preoperative clinical photograph, occlusal view, **j** preoperative clinical photograph, frontal view, **k** 3 months postoperative clinical photograph showing soft tissue contour, **l** 3 months postoperative clinical photograph showing IDP heights and vertical tissue level, **m** 6 months postoperative clinical photograph showing soft tissue contour, **n** 6 months postoperative clinical photograph showing IDP heights and vertical tissue level, **o** 0–6 months superimposed models by best fit algorithm, and **p** 0–6 months superimposed models showing loss of 3.14 mm in buccal soft tissue contour

Table 1 shows volumetric changes recorded for both groups throughout the study. The presented results within the SCTG group showed that there was a statistically significant difference (*P* < 0.05) between different follow-up periods. Similarly, the minimally-traumatic extraction followed by spontaneous healing group demonstrated a statistically significant difference (*P* < 0.05) in the mean change of buccal soft tissue contour between follow-up periods. Intergroup analysis between the two modalities at 1.5 mm, 3 mm, and 4.5 mm revealed statistically significant difference from 0 to 3 months and 0 to 6 months (*P* < 0.001). However, there was no statistically significant difference at the same 3 measured points from 3 to 6 months (*P* > 0.05). Interestingly, sites treated with SCTG showed a statistically significant (*P* < 0.001) gain in mean buccal soft tissue contour compared to atraumatic extraction group after 6 months (Fig. 3).

**Table 1:** Volumetric change of buccal soft tissue contour and clinical parameters of both studied groups throughout the experimental period

Level of measurement from FGM	Time interval	Volumetric change of buccal soft tissue contour (mm)		Mean difference [95% CI]	*P* value
		STCG Mean (± SD)	Minimally-traumatic extraction + spontaneous healing Mean (± SD)		
1.5 mm	0–3	− 0.38 ± 0.31	− 2.05 ± 0.81	1.66 [1.23, 2.09]	*P* < 0.001*
	0–6	− 0.53 ± 0.40	− 2.17 ± 0.80	1.64 [1.20, 2.09]	*P* < 0.001*
	3–6	− 0.14 ± 0.21	− 0.12 ± 0.28	1.20 [0.84, 1.55]	*P* = 0.837
3 mm	0–3	0.07 ± 0.31	− 1.47 ± 0.66	1.54 [1.18, 0.91]	*P* < 0.001*
	0–6	− 0.11 ± 0.44	− 1.73 ± 0.63	1.61 [1.22, 1.99]	*P* < 0.001*
	3–6	− 0.19 ± 0.24	− 0.26 ± 0.41	0.06 [− 0.17, 0.30]	*P* = 0.580
4.5 mm	0–3	0.30 ± 0.39	− 1.12 ± 0.57	1.43 [1.09, 1.77]	*P* < 0.001*
	0–6	0.14 ± 0.50	− 1.34 ± 0.69	1.48 [1.06, 1.90]	*P* < 0.001*
	3–6	− 0.17 ± 0.30	− 0.22 ± 0.34	0.05 [− 0.17, 0.27]	*P* = 0.639
Level of measurement from FGM	Time interval	Gingival thickness (mm)		Mean difference [95% CI]	*P* value
		STCG Mean (± SD)	Minimally-traumatic extraction + spontaneous healing Mean (± SD)		
1.5 mm	Baseline	1.26 ± 0.39	1.23 ± 0.46	0.02 [− 0.25, 0.30]	*P* = 0.831
	3 months	5.26 ± 1.49	5.23 ± 1.99	0.02 [− 1.20, 1.25]	*P* = 0.961
	6 months	3.52 ± 1.17	1.23 ± 0.43	2.29 [1.67, 2.91]	*P* < 0.001*
3 mm	Baseline	1.26 ± 0.53	1.5 ± 0.68	− 0.23 [− 0.66, 0.19]	*P* = 0.272
	3 months	4.88 ± 1.37	3.05 ± 1.58	1.82 [0.78, 2.86]	*P* < 0.001*
	6 months	3.14 ± 0.99	0.88 ± 0.41	2.26 [1.73, 2.79]	*P* < 0.001*
4.5 mm	Baseline	0.79 ± 0.39	0.82 ± 0.39	− 0.02 [− 0.30, 0.24]	*P* = 0.829
	3 months	2.58 ± 1.18	1.17 ± 0.58	1.41 [0.75, 2.06]	*P* < 0.001*
	6 months	2.20 ± 0.88	0.67 ± 0.24	1.52 [1.07, 1.98]	*P* < 0.001*
Site	Time interval	IDP height (mm)		Mean difference [95% CI]	*P* value
		STCG Mean (± SD)	Minimally-traumatic extraction + spontaneous healing Mean (± SD)		
MIP	Baseline	2.76 ± 0.43	2.67 ± 0.70	0.08 [− 0.32, 0.49]	*P* = 0.664
DIP		2.47 ± 0.37	2.55 ± 0.46	− 0.08 [− 0.38, 0.20]	*P* = 0.545
MIP	3 months	2.55 ± 0.68	1.9 ± 0.42	0.61 [0.22, 1.01]	*P* = 0.003*
DIP		2.29 ± 0.43	1.85 ± 0.38	0.44 [0.15, 0.72]	*P* = 0.003*
MIP	6 months	2.47 ± 0.51	1.61 ± 0.37	0.85 [0.53, 1.16]	*P* < 0.001*
DIP		2.29 ± 0.30	1.61 ± 0.33	0.67 [0.45, 0.90]	*P* < 0.001*

*Corresponds to statistically significant difference

#### Vertical tissue loss

The present results showed that the SCTG group presented less vertical tissue loss with mean (± SD) of − 0.66 (± 0.62), while the minimally-traumatic extraction followed by spontaneous healing group showed − 1.75 (± 0.73) with a statistically significant difference (*P* < 0.001) between them from 0 to 6 months postoperatively.

### Clinical outcomes

#### Gingival thickness

Table 1 shows GT recorded for both groups throughout the study. Both groups showed the same mean baseline values for GT at 1.5 mm, 3 mm, and 4.5 mm. There was a statistically significant (*P* < 0.001) increase in the mean GT at 3 and 6 months postoperatively compared to baseline values in the SCTG group. Meanwhile, the minimally-traumatic extraction followed by spontaneous healing group presented a statistically significant (*P* < 0.05) decrease in mean GT at 3 and 6 months postoperatively compared to baseline values. The STCG group showed a statistically significant increase in mean GT at 1.5 mm, 3 mm, and 4.5 mm (*P* < 0.001) when compared to minimally-traumatic extraction followed by spontaneous healing group after 6 months.

#### Interdental papilla height

Table 1 shows IDP heights recorded for both groups throughout the study. This investigation observed that there was no statistically significant difference in both mesial IDP (*P* = 0.664) and distal IDP (*P* = 0.545) heights between the two groups at baseline, yet at 3 and 6 months there was a statistically significant difference (*P* < 0.05) in both mesial and distal IDP heights between both groups.

## Discussion

Several techniques were suggested for alveolar ridge contour preservation including immediate implant placement, socket grafting, guided tissue regeneration concept, platelets concentrate, or other protocols like socket seal surgery and partial extraction therapy [2, 15, 25–27]. Despite their effectiveness and predictability, there are still few drawbacks regarding the esthetic outcome, since they mainly focus on hard tissue regeneration [28–30]. Furthermore, none of the proposed protocols could entirely avoid the soft tissue remodeling phenomenon, even some cases resulted in an esthetic discrepancy with an observed buccal concavity [31–33]. With regard to the above-mentioned gap of knowledge, the present trial targeted only the impact of soft tissue augmentation by SCTG on the post-extraction volumetric changes of buccal soft tissue contour.

Soft tissue augmentation techniques, such as bilaminar technique/coronally advanced flap and the pouch procedure with CTG, have emerged to address esthetic concerns in previous techniques. Such protocols showed predictability and improved soft tissue dehiscence and volume loss around dental implants [34–36]. Moreover, Marzadori et al. [37] concluded that the pouch technique with SCTG is the ultimate choice for soft tissue augmentation, particularly in areas of high esthetic demands, maintaining color and soft tissue appearance. To the best of the authors’ knowledge, this study is the first randomized controlled clinical trial comparing minimally-traumatic extraction with SCTG to minimally-traumatic extraction followed by spontaneous healing, investigating the influence of soft tissue management in fresh extraction sockets using volumetric analysis. No socket sealing material was used in the present study, in order to allow the inflammatory phase of the socket healing to begin [38], without any factors that might influence the healing process. Thus, this randomized clinical trial investigated the influence of soft tissue management solely in fresh extraction sockets via volumetric analysis without any confounding factors.

Post-extraction volumetric buccal soft tissue change is of utmost clinical interest, since the loss of root convexity may lead to unfavorable esthetic results and requires additional augmentation procedures. Accordingly, linear volumetric change of buccal soft tissue contour was considered the primary outcome in this study together with vertical tissue loss, gingival thickness, and interdental papilla height. A 6-month postoperative evaluation period was chosen based on the recommendation that final restorative measures should not be initiated until 6 months. Furthermore, qualitative and probably quantitative alterations can arise during the healing period of the augmented soft tissue [21, 22]. The accuracy of volume measurement with optical scanning-based digital technologies showed tremendous precision and high reproducibility [39]. Consequently, digitalized volumetric analysis was used to assess the linear volumetric change of buccal soft tissue contour and vertical tissue loss in the present clinical trial.

The findings presented herein showed that minimally-traumatic extraction followed by spontaneous healing without soft tissue augmentation caused a significant decrease in the buccal soft tissue contour, vertical tissue level, gingival thickness, and interdental papillae height after 6 months compared to baseline. These findings align with previous studies investigating spontaneous socket healing [40, 41]. Schneider et al. [40] observed buccal contour loss in spontaneously healing sites compared to sites treated with ridge preservation techniques. This was also supported by Thoma et al. [41] who reported a buccal soft tissue collapse of 1.2–1.6 mm in unassisted healing sites. The current study confirms the unsatisfactory esthetic results in unassisted socket healing presented in the literature [42, 43]. However, Clementini et al. [44] reported an increase in buccal soft tissue profile and thickness in unassisted healing sites after 4 months, attributing it to spontaneous soft tissue thickening phenomena. Similarly, Chappuis et al. [45] observed spontaneous soft tissue thickening in thin bone phenotypes after 8 weeks. This discrepancy might be attributed to the fact that the current investigation evaluated the outcomes after 6 months, representing the full maturation of hard and soft tissues at the extraction site [38]. Regarding the interdental papilla height, the minimally-traumatic extraction followed spontaneous healing group showed decreased IDP heights over time, likely due to the thin phenotype of the treated sites. Previous studies on papilla fill around implant restorations supported these results, indicating that gingival phenotype influenced papilla volume/fill [46–48].

On the other hand, results of this clinical trial showed that the use of SCTG buccal to extraction sockets significantly preserved buccal soft tissue contour, reduced vertical tissue loss, increased gingival thickness, and maintained interdental papillae height after 6 months. These findings were consistent with previous studies showing the benefits of SCTG in improving soft tissue profiles and correcting alveolar ridge contour defects [22, 24, 34, 49–51]. Furthermore, utilizing SCTG with a pouch preparation buccal to fresh extraction sockets limited post-extraction soft tissue alterations and yielded positive outcomes. These observations were supported by a recent systematic review by Zucchelli et al. [16], which emphasized the esthetic improvement and long-term stability achieved with SCTG in managing ridge deformities in the esthetic zone. The volumetric buccal soft tissue changes from baseline to 6 months in the SCTG group revealed a mean ± SD change of − 0.53 ± 0.40, − 0.11 ± 0.44, and 0.14 ± 0.50 mm at 1.5 mm, 3 mm, and 4.5 mm from the free gingival margin, respectively. These results were superior to the use of “saddled” connective tissue graft combined with biomaterials [34] and were inferior to those reported by De Bruyckere et al. [24] who compared SCTG with GBR to reestablish ridge profile at the buccal aspect of single implants. These differences might be attributed to the different timing of interventions and prosthetic involvement in shaping the augmented soft tissue. It is worthy to mention that the baseline assessment in this investigation was performed with the presence of the natural tooth transgingival support. Besides, no soft tissue shaping occurred during the follow-up period. Remarkably, the currently observed findings might suggest that the use of the pouch and SCTG technique might conceal the post-extraction buccal concavity.

The current statistical analysis revealed that all the outcomes measured in the minimally-traumatic extraction with SCTG group surpassed those of the minimally-traumatic extraction followed spontaneous healing group solely with no soft tissue augmentation after 6 months. These observations suggest that the presence of the SCTG might effectively increase the soft tissue volume and mask the post-extraction contour deficiencies, especially in the esthetic zone. Furthermore, the increased gingival thickness observed after the use of SCTG might imply its ability to act as a biologic filler, improve the stability of the interdental tissues, and enhance the gingival phenotype. The current data sheds light on the effectiveness of the pouch and SCTG technique in preserving alveolar ridge contour during post-extraction modeling and remodeling. Future studies are warranted to explore the influence of intraoral scanners on volumetric analysis, in addition to examining the use of prosthetic devices and their impact on soft tissue shaping after augmentation.

One of the limitations of the present clinical trial was that a third arm of bone graft placement into the socket as well as connective tissue graft should have been performed. Another limitation was that IDP measurements were not calibrated.

## Conclusions

Within the limitations of this randomized clinical trial study, it can be concluded that the pouch technique utilizing a SCTG buccal to fresh extraction sockets might be considered as an effective modality for alveolar ridge contour preservation and ridge profile stabilization up to 6 months. This study paves the way for future randomized clinical trials with longer follow-up periods.

## Data Availability

Data is available by contacting the author upon request.

## Footnotes

1 OnDemand3D software, Cybermed, Seoul, Korea.

2 The Arab Drug Company for pharmaceutical and Chemical Industries CO., Cairo, Egypt.

3 Hydrorise Putty and light, additional silicone, Zhermack SpA, Italy.

4 Shape lab scanner E series, Copenhagen, Denmark.

5 Exo cad, Fraunhofer Institute, Germany.

6 Helmut Zepf, Germany.

7 Kiato™ 15c surgical blade, Kiato, Germany.

8 Tunneling Instrument, #1, Helmut Zepf, Germany.

9 Ibuprofen 600 mg Abbott, Egypt.

10 Amoxicillin 500 mg Cap., Egyptian Int. Pharmaceutical Industrial Co., A. R. E.

11 Power and sample size program: biostat.mc.vanderbilt.edu/twiki/bin/view/Main/Power Sample Size.

12 MedCalc Software Ltd, Ostend, Belgium.
